# ﻿A new species of the *Cyrtodactylusbrevipalmatus* group (Squamata, Gekkonidae) from the uplands of western Thailand

**DOI:** 10.3897/zookeys.1141.97624

**Published:** 2023-01-19

**Authors:** L. Lee Grismer, Attapol Rujirawan, Siriwadee Chomdej, Chatmongkon Suwannapoom, Siriporn Yodthong, Akrachai Aksornneam, Anchalee Aowphol

**Affiliations:** 1 Herpetology Laboratory, Department of Biology, La Sierra University, 4500 Riverwalk Parkway, Riverside, California 92505, USA La Sierra University Riverside United States of America; 2 Department of Herpetology, San Diego Natural History Museum, PO Box 121390, San Diego, California 92112, USA San Diego Natural History Museum San Diego United States of America; 3 Animal Systematics and Ecology Speciality Research Unit, Department of Zoology, Faculty of Science, Kasetsart University, Bangkok 10900, Thailand Kasetsart University Bangkok Thailand; 4 Department of Biology, Faculty of Science, Chiang Mai University, Chiang Mai 50200, Thailand Chiang Mai University Chiang Mai Thailand; 5 Division of Fishery, School of Agriculture and Natural Resources, University of Phayao, Phayao 56000, Thailand University of Phayao Phayao Thailand; 6 Department of Biology, Faculty of Science, Thaksin University, Pa Phayom, Phattalung 93210, Thailand Thaksin University Phattalung Thailand

**Keywords:** Bent-toed gecko, genetics, Indochina, integrative taxonomy, montane forests, morphology

## Abstract

An integrative systematic analysis recovered a new species of the *Cyrtodactylusbrevipalmatus* group from the uplands of Thong Pha Phum National Park, Kanchanaburi Province in western Thailand. *Cyrtodactylusthongphaphumensis***sp. nov.** is deeply embedded within the *brevipalmatus* group, bearing an uncorrected pairwise sequence divergence of 7.6–22.3% from all other species based on a 1,386 base pair segment of the mitochondrial NADH dehydrogenase subunit 2 gene (ND2) and adjacent tRNAs. It is diagnosable from all other species in the *brevipalmatus* group by statistically significant mean differences in meristic and normalized morphometric characters as well as differences in categorical morphology. A multiple factor analysis recovered its unique and non-overlapping placement in morphospace as statistically significantly different from that of all other species in the *brevipalmatus* group. The description of this new species contributes to a growing body of literature underscoring the high degree of herpetological diversity and endemism across the sky-island archipelagos of upland montane tropical forest habitats in Thailand, which like all other upland tropical landscapes, are becoming some of the most imperiled ecosystems on the planet.

## ﻿Introduction

The gekkonid genus *Cyrtodactylus* Gray, 1827 contains well over 350 named and unnamed species and constitutes the third largest vertebrate genus on the planet (Grismer et al. 2021a, b; Uetz et al. 2022). To date, its extensive distribution extends across at least eight biogeographic regions and crosses a number of well-established biogeographic barriers from South Asia to western Melanesia (Grismer et al. 2022a). The ecological plasticity, phylogenetic relationships, and geographic distribution among, and within its 32 geographically circumscribed monophyletic species groups, are indicative of its ability to disperse across ephemeral seaways, major river systems, basins, mountain ranges, and land bridges, followed by extensive in situ diversification within specific geographic areas (Grismer et al. 2020, 2021a, b, 2022a).

**Figure 1.**
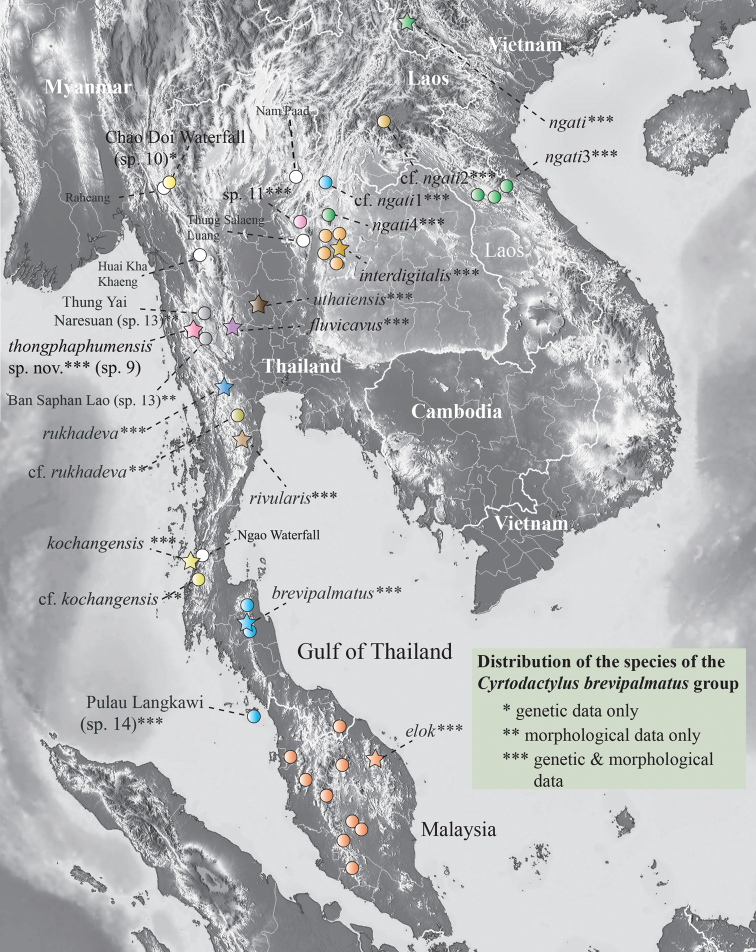
Distribution of nominal species and unnamed populations of the *Cyrtodactylusbrevipalmatus* group. Stars denote type localities. White circles are literature localities from which specimens were not examined and remain unidentified. Locality data for all material examined is in Grismer et al. (2022c: table 1).

Within Indochina and northern Sundaland, the *Cyrtodactylusbrevipalmatus* group is one of the most ecologically and morphologically specialized groups within *Cyrtodactylus* (sec. Grismer et al. 2020, 2021a, b). All members bear a similar morphology, behavior, and color pattern adapted to an arboreal life style (Grismer et al. 2022b). The latest phylogenetic taxonomic treatment of the group (Grismer et al. 2022c) described four new species from Thailand, resulting in ten described and potentially as many undescribed populations needing further study. One of these undescribed populations, *C.* sp. 9 from Thong Pha Phum National Park, Kanchanaburi Province in western Thailand (Fig. 1), was first recognized on the basis of molecular phylogenetic evidence from a single specimen (Chomdej et al. 2021). We collected and sequenced eight additional specimens which corroborate the results of Chomdej et al. (2021) in that all eight specimens plus the specimen of Chomdej et al. (2021) form a monophyletic lineage deeply nested within the *brevipalmatus* group (Grismer et al. 2022c). Univariate and multivariate analyses of the eight new specimens recovered statistically significant morphological and morphospatial differences from all other members of the group which unequivocally indicate that it requires species-level recognition (Grismer et al. 2022c). As such, it is described herein.

## ﻿Materials and methods

### ﻿Genetic data

Methods for DNA extraction, sequencing, and editing followed Grismer et al. (2021c) and resulted in a 1,386 base pair segment of the mitochondrial NADH dehydrogenase subunit 2 gene (ND2) and adjacent tRNAs. All material examined is listed in Grismer et al. (2022c: table 1) along with GenBank accession numbers.

### ﻿Morphological data

The morphological data taken included 17 meristic, 18 normalized morphometric, and eight categorical characters (Grismer et al. 2022c) (Table 1). Normalization of the morphometric characters followed the method of Chan and Grismer (2022).

**Table 1.** Descriptions of morphometric, meristic, and categorical characters.

Abbreviations	Characters
**Morphometric characters**	
**SVL**	snout-vent length, taken from the tip of the snout to the vent
**TL**	tail length, taken from the vent to the tip of the tail–original or partially regenerated
**TW**	tail width, taken at the base of the tail immediately posterior to the postcloacal swelling
**HumL**	humeral length, taken from the proximal end of the humerus at its insertion point in the glenoid fossa to the distal margin of the elbow while flexed 90°
**ForL**	forearm length, taken on the ventral surface from the posterior margin of the elbow while flexed 90° to the inflection of the flexed wrist
**FemL**	femur length, taken from the proximal end of the femur at its insertion point in the acetabulum to the distal margin of the knee while flexed 90°
**TibL**	tibia length, taken on the ventral surface from the posterior margin of the knee while flexed 90° to the base of the heel
**AG**	axilla to groin length, taken from the posterior margin of the forelimb at its insertion point on the body to the anterior margin of the hind limb at its insertion point on the body
**HL**	head length, the distance from the posterior margin of the retroarticular process of the lower jaw to the tip of the snout
**HW**	head width, measured at the angle of the jaws
**HD**	head depth, the maximum height of head measured from the occiput to base of the lower jaw posterior to the eyes
**ED**	eye diameter, the greatest horizontal diameter of the eye-ball
**EE**	eye to ear distance, measured from the anterior edge of the ear opening to the posterior edge of the bony orbit
**ES**	eye to snout distance or snout length, measured from anteriormost margin of the bony orbit to the tip of snout
**EN**	eye to nostril distance, measured from the anterior margin of the bony orbit to the posterior margin of the external nares
**IO**	interorbital distance, measured between the dorsomedial-most edges of the bony orbits
**IN**	internarial distance, measured between the external nares across the rostrum
**EL**	ear length, greatest oblique length across the auditory meatus.
**Meristic characters**	
**SL**	supralabial scales, counted from the largest scale at the corner of the mouth or posterior to the eye, to the rostral scale
**IL**	infralabial scales, counted from termination of enlarged scales at the corner of the mouth to the mental scale
**PVT**	paravertebral tubercles between the limb insertions, counted in a straight line immediately left of the vertebral column
**LRT**	longitudinal rows of body tubercles, counted transversely across the body midway between the limb insertions from one ventrolateral body fold to the other
**VS**	longitudinal rows of ventral scales, counted transversely across the abdomen midway between limb insertions from one ventrolateral fold to the other
**VSM**	transverse rows of ventral scales, counted along the midline of the body from the postmentals to just anterior to the cloacal opening, stopping where the scales become granular
**TL4E**	expanded subdigital lamellae on the fourth toe proximal to the digital inflection, counted from the base of the first phalanx where it contacts the body of the foot to the largest scale on the digital inflection–the large contiguous scales on the palmar and plantar surfaces were not counted
**TL4U**	small, generally unmodified subdigital lamellae distal to the digital inflection on the fourth toe, counted from the digital inflection to the claw including the claw sheath
**TL4T**	total number of subdigital lamellae beneath the fourth toe, TL4E + TL4U = TL4T
**FL4E**	number of expanded subdigital lamellae on the fourth finger proximal to the digital inflection, counted the same way as with TL4E
**FL4U**	small generally unmodified subdigital lamellae distal to the digital inflection on the fourth finger, counted the same way as with TL4U
**FL4T**	total number of subdigital lamellae beneath the fourth toe, FL4E + FL4U = FL4T
**FS**	enlarged femoral scales, counted from each thigh and combined as a single metric
**PCS**	enlarged precloacal scales, counted as a single metric
**PP**	number of precloacal pores in males, counted as a single metric
**FP**	femoral pores in males, counted from each thigh and combined as a single metric
**BB**	number of dark body bands, counted from between the dark band on the nape and the hind limb insertions on the body
**Categorical characters**	
**FKT**	tubercles on the flanks (present or absent)
**SC1**	slightly enlarged medial subcaudals (present or absent)
**SC2**	single distinctly enlarged, unmodified, row of medial subcaudal scales (present or absent)
**SC3**	enlarged medial subcaudals intermittent, medially furrowed, posteriorly emarginated (yes or no)
**DCT**	dorsolateral caudal tubercles (small or large)
**VLF1**	DCT forming a ventrolateral caudal fringe (narrow or wide)
**VLF2**	ventrolateral caudal fringe scales generally homogenous or not (yes or no)
**TLcross**	cross-section of the tail (round or square)

### ﻿Phylogenetic analyses

Following Grismer et al. (2022c), an input file implemented in BEAUti (Bayesian Evolutionary Analysis Utility) v. 2.4.6 was run in BEAST (Bayesian Evolutionary Analysis Sampling Trees) v. 2.4.6 (Drummond et al. 2012) on CIPRES (Cyberinfrastructure for Phylogenetic Research; Miller et al. 2010) in order to generate a BEAST phylogeny, employing a lognormal relaxed clock with unlinked site models and linked trees and clock models. bModelTest (Bouckaert and Drummond 2017), implemented in BEAST, was used to numerically integrate over the uncertainty of substitution models while simultaneously estimating phylogeny using Markov chain Monte Carlo (MCMC). MCMC chains were run using a Yule prior for 40,000,000 million generations and logged every 4,000 generations. The BEAST log file was visualized in Tracer v. 1.7.0 (Rambaut et al. 2018) to ensure effective sample sizes (ESS) were well-above 200 for all parameters. A maximum clade credibility tree using mean heights at the nodes was generated using TreeAnnotator v. 1.8.0 (Rambaut and Drummond 2013) with a burn-in of 1,000 trees (10%). Nodes with Bayesian posterior probabilities (BPP) of 0.95 and above were considered strongly supported (Huelsenbeck et al. 2001; Wilcox et al. 2002). Uncorrected pairwise sequence divergences were calculated in MEGA 11 (Tamura et al. 2021) using the complete deletion option to remove gaps and missing data from the alignment prior to analysis.

### ﻿Statistical analyses

All statistical analyses were conducted using R Core Team (2018). A Levene’s test for the normalized morphometric and meristic characters was conducted to test for equal variances across all groups. Characters with equal variances (*F* ≥ 0.05) were analyzed by an analysis of variance (ANOVA) and TukeyHSD post hoc test. Those with unequal variances (*F* < 0.05) were subjected to Welch’s F-test and Games-Howell *post hoc* test.

Morphospatial clustering and positioning among the species was analyzed using multiple factor analysis (MFA) on a concatenated data set comprised of 38 characters including non-metric categorical characters which cannot be used in a principal component analysis (Suppl. material 1). The MFA was implemented using the mfa() command in the R package FactorMineR (Husson et al. 2017) and visualized using the Factoextra package (Kassambara and Mundt 2017). A non-parametric permutation multivariate analysis of variance (PERMANOVA) from the *vegan* package 2.5–3 in R (Oksanen et al. 2020) was used to determine the statistical significance of centroid locations and group clustering. The analysis used a Euclidean (dis)similarity matrix with 50,000 permutations based on the loadings of the first four dimensions recovered from the MFA. The highly morphologically derived *Cytodactyluselok* was not included so as to prevent biasing the morphospatial relationships among the other species (see Grismer et al. 2022b).

## ﻿Results

### ﻿Phylogenetic analysis

**Figure 2.**
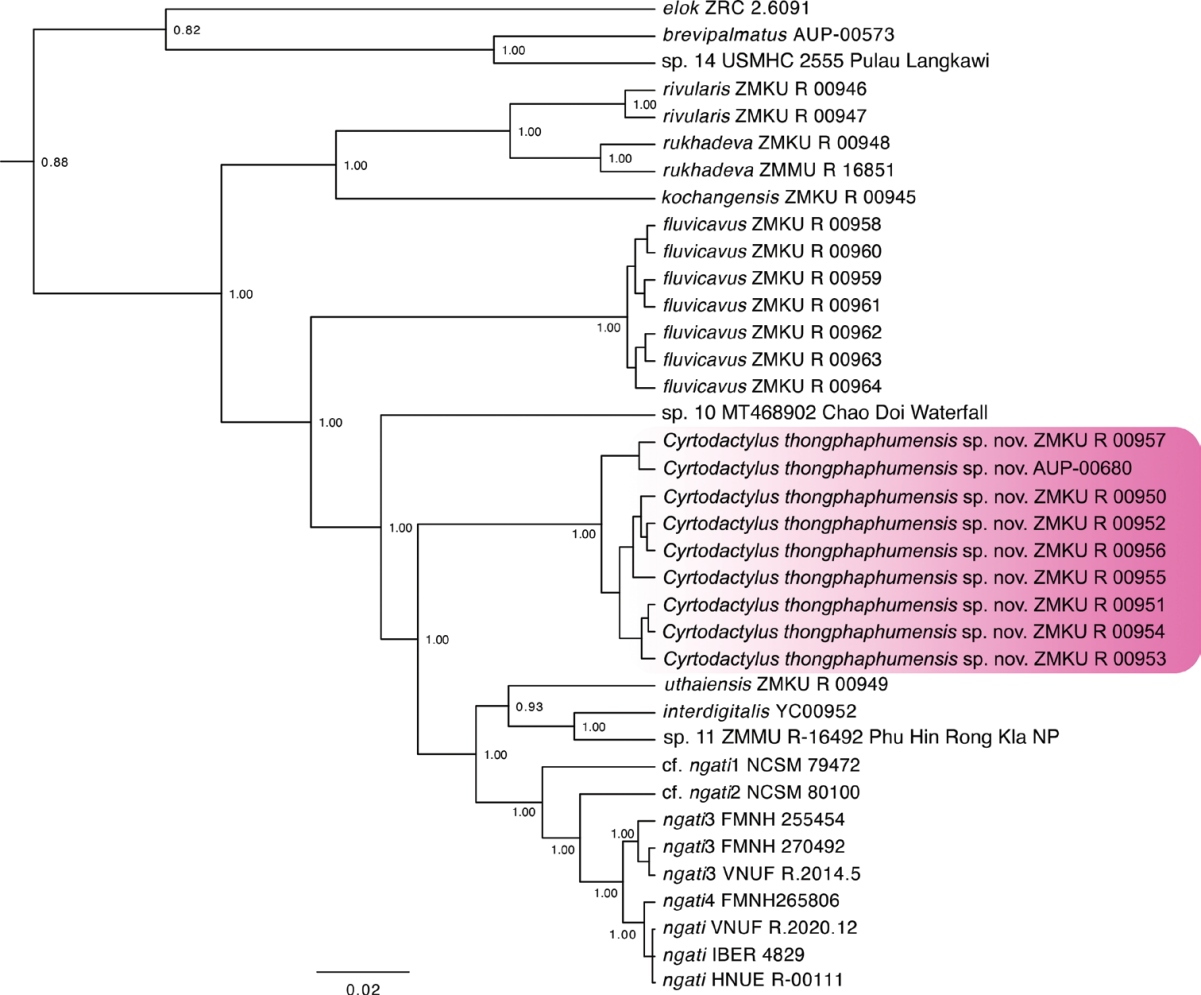
Maximum clade credibility BEAST phylogeny of the *Cyrtodactylusbrevipalmatus* group highlighting the new species described herein. Bayesian posterior probabilities (BPP) are listed at the nodes.

The BEAST analysis recovered the Thong Pha Phum population as being deeply embedded within the *brevipalmatus* group and the strongly supported (1.00) sister lineage to two sister groups composed of (1) *C.interdigitalis*, *C.uthaiensis*, and *C.* sp. 11 and (2) C.cf.ngati1, C.cf.ngati2, *C.ngati*3, *C.ngati*4, and *C.ngati* (Fig. 2). The uncorrected pairwise sequence divergence between the Thong Pha Phum population and all other species of the *brevipalmatus* group ranges from 7.6–22.3%. (Table 2).

**Table 2.** Mean (minimum–maximum) percentages of uncorrected pairwise sequence divergence (*p*-distances) among the putative species of the *Cyrtodactylusbrevipalmatus* group based on 1,386 base pairs of mitochondrial NADH dehydrogenase subunit 2 gene (ND2) and adjacent tRNAs. Intraspecific p-distance are in bold font. n/a = data not applicable.

Species	1. *C.brevipalmatus*	2. C.cf.ngati1	3. C.cf.ngati2	4. *C.elok*	5. *C.fluvicavus*	6. *C.interdigitalis*	7. *C.kochangensis*	8. *C.ngati*, *C.ngati*3 and *C.ngati*4	9. *C.rivularis*	10. *C.rukhadeva*	11. *C.thongphaphumensis* sp. nov	12. *C.* sp. 10	13. *C.* sp. 11	14. *C.* sp. 14	15. *C.uthaiensis*
***N***	1	1	1	1	7	1	1	7	2	2	9	1	1	1	1
1.	**n/a**														
2.	21.03	**n/a**													
3.	21.68	4.39	**n/a**												
4.	20.77	22.58	21.42	**n/a**											
5.	18.86 (18.84–18.97)	10.64 (10.58–10.84)	11.02 (10.97–11.23)	20.15 (20.13–20.26)	**0.10 (0.00–0.26)**										
6.	20.77	6.97	9.16	22.84	12.02 (12.00–12.13)	**n/a**									
7.	19.35	14.58	14.71	20.90	12.31 (12.26–12.31)	15.23	**n/a**								
8.	20.70 (20.65–20.90)	3.30 (2.84–4.00)	3.71 (3.35–4.26)	21.11 (20.90–21.42)	11.34 (11.10–11.87)	8.13 (7.74–8.65)	14.58 (14.45–14.84)	**0.84 (0.00–1.55)**							
9.	20.00 (19.74–20.26)	15.87 (15.61–16.13)	15.03 (14.84–15.23)	21.61 (21.42–21.81)	12.57 (12.26–13.03)	15.48 (15.23–15.74)	12.26 (12.00–12.52)	15.03 (14.71–15.48)	**0.52**						
10.	20.65 (20.13–21.16)	15.42 14.84–16.00)	15.48 (14.84–16.13)	21.61 (21.16–22.06)	12.25 (11.61–13.03)	16.00 (15.35–16.65)	13.10 (12.52–13.68)	15.23 (14.19–16.23)	4.65 (3.61–5.68)	**1.55**					
11.	20.34 (20.13–20.65)	7.93 (7.74–8.00)	9.51 (9.42–9.55)	22.02 (21.81–22.32)	9.75 (9.55–9.94)	8.96 (8.77–9.03)	13.22 (13.03–13.29)	8.81 (8.13–9.68)	13.12 (12.77–13.42)	13.25 (12.52–13.94)	**0.22 (0.00–0.52)**				
12.	19.87	9.29	10.84	21.94	10.12 (10.06–10.32)	10.19	13.68	10.21 (10.06–10.45)	13.94 (13.68–14.19)	14.32 (13.68–14.97)	8.06 (7.87–8.13)	**n/a**			
13.	20.39	7.23	8.90	22.19	11.12 (11.10–11.23)	3.87	14.58	8.28 (8.00–8.65)	15.35 (15.10–15.61)	15.61 (14.97–16.26)	8.96 (8.77–9.03)	10.45	**n/a**		
14.	6.45	20.90	20.65	20.00	18.34 (18.32–18.45)	20.13	19.10	20.52 (20.26–20.65)	19.74 (19.48–20.00)	20.00 (19.48–20.52)	19.60 (19.48–19.87)	18.84	19.61	**n/a**	
15.	19.74	5.81	8.13	21.16	10.12 (10.06–10.32)	7.1	13.94	6.97 (6.58–7.61)	13.94 (13.68–14.19)	13.94 (13.29–14.58)	7.80 (7.61–7.87)	8.39	6.58	19.48	**n/a**

### ﻿Statistical analyses

**Figure 3.**
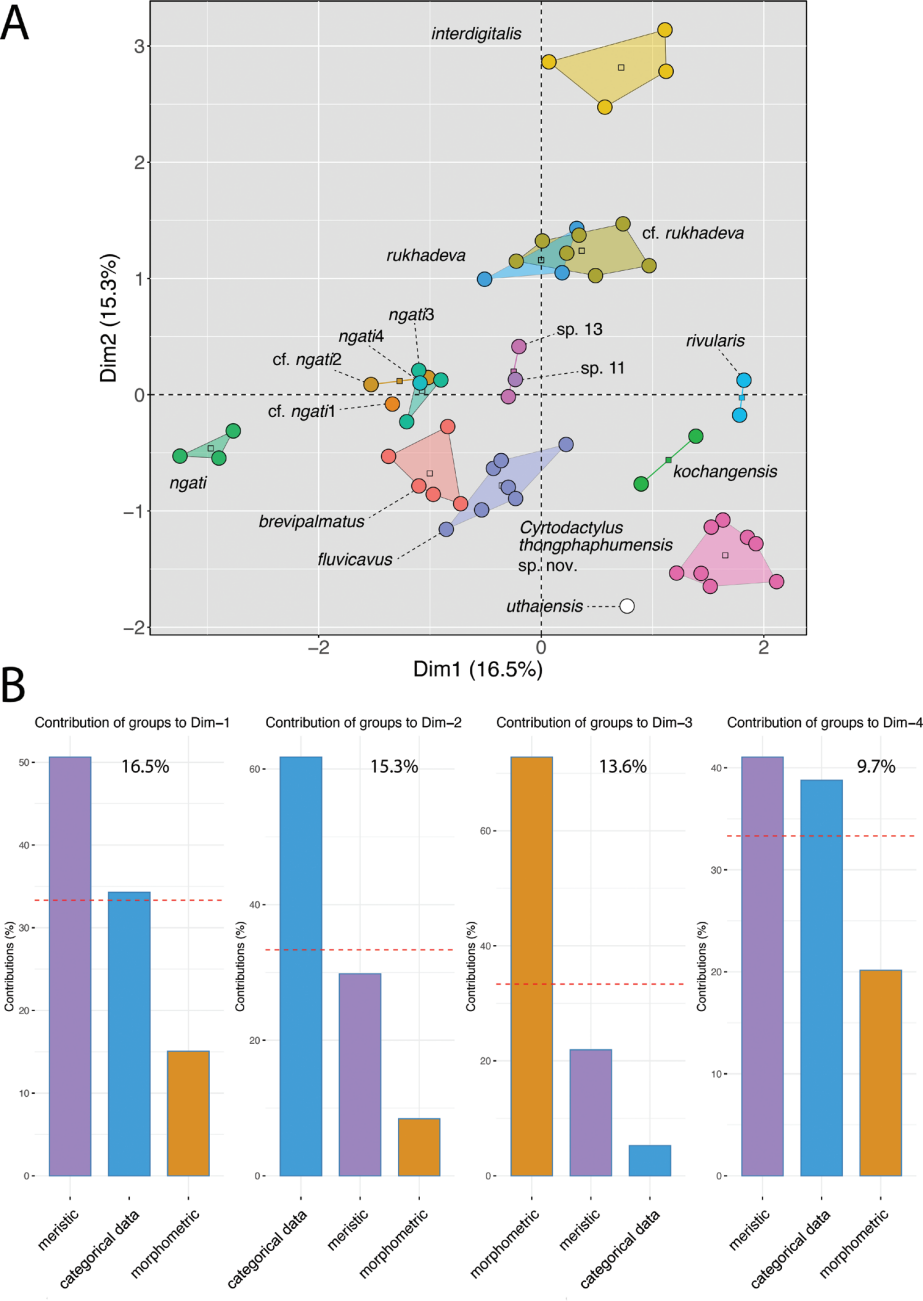
**A**MFA of the species-level lineages based on the BEAST phylogeny (Fig. 2) **B** Percent contributions of each data type to the inertia of dimensions 1–4 of the MFA. Percentage values on the bar graphs are the amounts of inertia for the respective dimensions.

The ANOVA and TukeyHSD *post hoc* and Welch’s F-test and Games-Howell post hoc tests of the adjusted morphometric and meristic characters were consistent with the phylogenetic and pairwise distance data in recovering a number of statistically significant differences between the Thong Pha Phum population and all other species (Table 3). Thong Pha Phum population plotted separately in the MFA with meristic data contributing 16.5% of the inertia in dimension 1, categorical morphology contributing 15.3% of the inertia in dimension 2, and normalized morphometric data contributing 13.6% of the inertia in dimension 3 (Fig. 3). The PERMANOVA analysis recovered the morphospatial position of the Thong Pha Phum population as being statistically different from *C.brevipalmatus*, C.cf.ngati2, *C.ngati*3, *C.ngati*, *C.fluvicavus*, *C.interdigitalis*, *C.rivularis*, *C.rukhadeva*, and *Cyrtodactylus* sp. 13 (Table 4).

**Table 3.** Significant *p*-values from the results of the ANOVA and Welch’s *F* (*) analyses comparing the normalized morphometric and meristic characters of *Cyrtodactylusthongphaphumensis* sp. nov. to other species of the *Cyrtodactylusbrevipalmatus* group. Only species with and *N* > 2 are included. No significant differences were recovered for SVL. Abbreviations are in the Materials and methods.

Morphometric characters	AG*	HumL*	ForL	FemL	TibL	HL	HW	HD*	ED*	EE*	ES	EN*	IO	EL	IN
*C.brevipalmatus*						0.01	< 0.001				< 0.001	0.03			
*C.fluvicavus*			0.0					0.007			0.013	0.023		0.007	
*C.interdigitalis*					0.00		0.007								
*C.ngati*	< 0.001						< 0.001	0.042			0.007	< 0.001		< 0.001	0.000
*C.ngati*3	0.001				0.01	0.03	0.003	0.043		0.001	0.019	0.019			0.003
*C.rukhadeva*	0.02	0.004			0.02			0.033							
**Meristic characters**	**SL**	IL*	PVT*	**LRT**	**VS**	**VSM**	**TL4E**	**TL4T**	**FL4E**	FL4U*	FL4T*	**FS**	PCS*	BB*	
*C.brevipalmatus*				< 0.001	0.003					0.022			< 0.001	0.05	
*C.fluvicavus*			< 0.001	< 0.001						0.001	0.004	0.020			
*C.interdigitalis*			0.003		< 0.001	0.005			0.043		0.01			< 0.001	
*C.ngati*	0.003		0.016	0.011				0.000	< 0.001	< 0.001			< 0.001	< 0.001	
*C.ngati*3			0.001	0.042						< 0.001	0.001				
*C.rukhadeva*	0.029		< 0.001		0.002									0.001	

**Table 4.** Summary statistics from the PERMANOVA analysis from the loadings of dimension 1–4 of the MFA comparing *Cyrtodactylusthongphaphumensis* sp. nov. to all other species the *Cyrtodactylusbrevipalmatus* group with sample sizes > 1. Bold fonts denote significant differences.

OTU pairs	F model	R^2^	*p*-value	*p*-adjusted
*C.rukhadeva*	88.504	0.847	**0.000**	**0.001**
C.cf.ngati2	56.471	0.876	**0.020**	1.000
*C.ngati*3	59.321	0.868	**0.006**	0.324
*C.interdigitalis*	85.773	0.896	**0.002**	0.112
*C.ngati*	134.367	0.937	**0.006**	0.332
*C.brevipalmatus*	80.229	0.879	**0.001**	**0.025**
*C.fluvicavus*	55.127	0.809	**0.000**	**0.008**
*C.rivularis*	9.485	0.542	**0.022**	1.000
*C.* sp. 13	30.716	0.793	**0.022**	1.000

## ﻿Taxonomy

Given the phylogenetic delimitation of the Thong Pha Phum population (Fig. 2), its statistically significant diagnostic morphological differences (Table 3), its statistically significant diagnostic placement in morphospace (Fig. 3, Table 4), and its notable difference in pairwise sequence divergence from all other species (Table 2), we describe it below as new species.

### ﻿ Cyrtodactylus thongphaphumensis sp. nov.

Taxon classificationAnimaliaSquamataGekkonidae

30D8F2B0-C45B-5C2E-8459-B4B93AE4106B

https://zoobank.org/4BB0E9B3-1BFF-49BC-BF77-79BF8CC95D27

**Figure 4.**
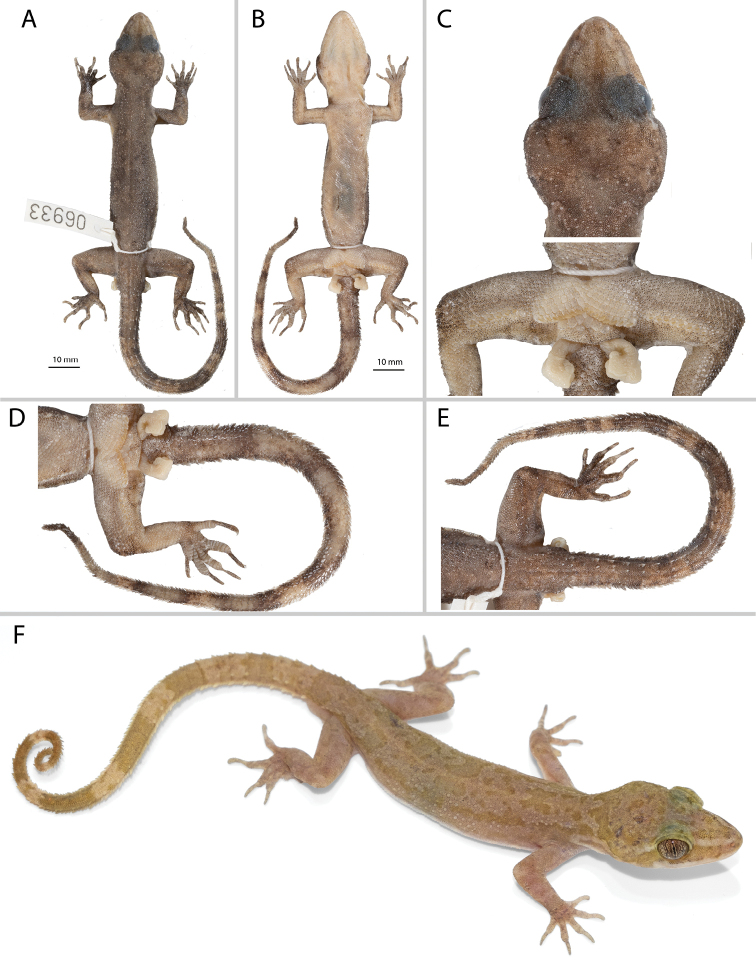
Adult male holotype of *Cyrtodactylusthongphaphumensis* sp. nov. ZMKU R 00953 (field no. AA 06933) from Thong Pha Phum National Park, Pilok Subdistrict, Thong Pha Phum District, Kanchanaburi Province, Thailand. **A** dorsal view **B** ventral view **C** dorsal view of head and ventral view of pelvic region **D** dorsal view of tail and **E** ventral view of tail in preservative **F** holotype in life.

**Figure 5.**
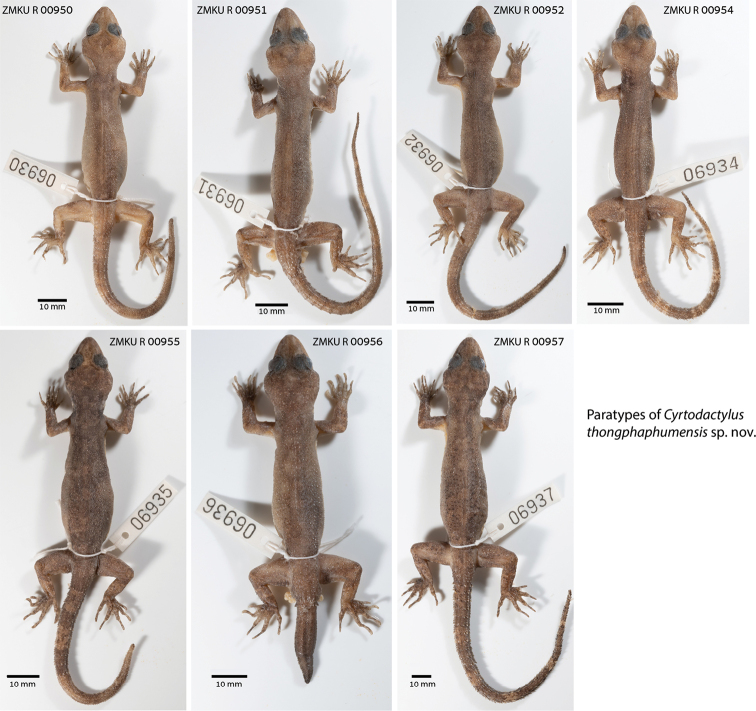
Paratypes of *Cyrtodactylusthongphaphumensis* sp. nov. in preservative from Thong Pha Phum National Park, Pilok Subdistrict, Thong Pha Phum District, Kanchanaburi Province, Thailand.

Suggested Common Name: Thong Pha Phum Bent-toed Gecko Figs 4 , 5

Cyrtodactylus sp. 9 Chomdej et al. 2021: 2; Grismer et al. 2022b: 248; Grismer et al. 2022c: 115.

#### Type material.

***Holotype*.** Adult male ZMKU R 00953 from Thong Pha Phum National Park, Pilok Subdistrict, Thong Pha Phum District, Kanchanaburi Province, Thailand (14.69339°N, 98.40534°E, 914 m a.s.l.), collected by Korkhwan Termprayoon, Akrachai Aksornneam, Natee Ampai, and Siriporn Yodthong on 8 April 2019.

***Paratypes*.** Adult males ZMKU R 00951, ZMKU R 00954 and ZMKU R 00956 and adult females ZMKU R 00950, ZMKU R 00952, ZMKU R 00955, and ZMKU R 00957 bear the same collection data as the holotype.

#### Diagnosis.

*Cyrtodactylusthongphaphumensis* sp. nov. can be separated from all other species of the *brevipalmatus* group by the combination of having 12–14 supralabials, 8–10 infralabials, 30–36 paravertebral tubercles, 19–21 rows of longitudinally arranged tubercles, 30–34 longitudinal rows of ventrals, 150–173 transverse rows of ventrals, 8–10 expanded subdigital lamellae on the fourth toe, 11–14 unexpanded subdigital lamellae on the fourth toe, 20–24 total subdigital lamellae on the fourth toe; seven or eight expanded subdigital lamellae on the fourth finger, 10–12 unexpanded subdigital lamellae on the fourth finger, 18–20 total subdigital lamellae on the fourth finger; 12–16 total number of enlarged femoral scales, 12–16 total number of femoral pores in males; 15 precloacal pores in males; 15–17 enlarged precloacals; enlarged femorals and enlarged precloacals not continuous; proximal femorals smaller than distal femorals; small tubercles on forelimbs and flanks; large dorsolateral caudal tubercles and wide ventrolateral caudal fringe; ventrolateral caudal fringe composed scales of different size; tail square in cross-section; maximum SVL 76.6 mm; 3–5 dark transverse body bands (Table 5).

**Table 5.** Sex and raw meristic, categorical, and morphometric data used in the analyses from specimens in the *Cyrtodactylusbrevipalmatus* group. Abbreviations: R/L = right/left; / = data unavailable.

**Species**	***Cyrtodactylusthongphaphumensis* sp. nov.**								***C.brevipalmatus***	**C.cf.brevipalmatus (*C.* sp. 14)**		***C.brevipalmatus***			
**Institutional catalog number**	**ZMKU R 00950 paratype**	**ZMKU R 00951 paratype**	**ZMKU R 00952 paratype**	**ZMKU R 00953 holotype**	**ZMKU R 00954 paratype**	**ZMKU R 00955 pratype**	**ZMKU R 00956 pratype**	**ZMKU R 00957 pratype**	**LSUHC 1899**	**LSUHC 15076**	**LSUHC 11788**	**THNHM 10670**	**THNHM 14112**		
**Sex**	♀	♂	♀	♂	♂	♀	♂	♀	♂	♀	♀	♀	♀		
**Meristic data**															
Supralabials (SL)	12	13	13	14	13	13	13	13	11	12	10	14	12		
Infralabials (IL)	8	8	10	10	9	10	10	9	8	10	9	11	11		
Paravertebral tubercles (PVT)	32	33	34	34	36	36	30	30	39	37	38	37	37		
Longitudinal rows of tubercles (LRT)	21	19	20	20	21	21	19	19	15	16	17	16	14		
Ventral scales (VS)	34	33	33	34	30	33	32	33	38	38	38	36	39		
Ventral scales along middle of the body (VSM)	173	158	156	166	159	159	150	169	176	170	182	154	160		
Expanded subdigital lamellae on 4^th^ toe (TL4E)	9	10	9	8	10	8	9	9	7	8	9	8	8		
Unmodified subdigital lamellae on 4^th^ toe (TL4U)	12	14	13	12	13	12	11	13	13	11	11	11	12		
Total subdigital lamellae 4^th^ toe (TL4T)	21	24	22	20	23	20	20	22	20	19	20	19	20		
Expanded subdigital lamellae on 4^th^ finger (FL4E)	8	7	7	8	8	8	8	8	8	8	8	7	8		
Unmodified subdigital lamellae on 4^th^ finger (FL4U)	10	12	12	11	12	12	11	12	9	11	10	10	10		
Total subdigital lamellae 4^th^ finger (FL4T)	18	19	19	19	20	20	19	20	17	19	18	17	18		
Enlarged femoral scales (R/L)	5R/7L	8R/8L	8R/8L	7R/8L	8R/8L	7R/8L	7R/6L	8R/8L	0	0	0	8R/8L	7R/7L		
Total enlarged femoral scales (FS)	12	16	16	15	16	15	13	16	16	10	11	16	14		
Total femoral pores in males (FP)	/	16	/	14	15	/	12	/	7	/	/	/	/		
Enlarged precloacal scales (PCS)	17	15	15	15	15	15	15	15	7	7	7	8	7		
Precloacal pores in males (PP)	/	15	/	15	15	/	15	/	7	/	/	/	/		
Postcloacal tubercles (PCT)	2	2R/3L	3	3	2R/3L	2R/3L	3	2	3	3	2	3	3		
Body bands (BB)	3	4	3	4	3	5	4	4	4	6	3	5	5		
**Species**	***Cyrtodactylusthongphaphumensis* sp. nov.**								***C.brevipalmatus***	**C.cf.brevipalmatus (*C.* sp. 14)**		***C.brevipalmatus***			
**Institutional catalog number**	**ZMKU R 00950 paratype**	**ZMKU R 00951 paratype**	**ZMKU R 00952 paratype**	**ZMKU R 00953 holotype**	**ZMKU R 00954 paratype**	**ZMKU R 00955 pratype**	**ZMKU R 00956 pratype**	**ZMKU R 00957 pratype**	**LSUHC 1899**	**LSUHC 15076**	**LSUHC 11788**	**THNHM 10670**	**THNHM 14112**		
**Sex**	♀	♂	♀	♂	♂	♀	♂	♀	♂	♀	♀	♀	♀		
**Categorical data**															
Small tubercles on flank (FKT)	present	present	present	present	present	present	present	present	present	present	present	present	present		
Dorsolateral caudal tubercles (DCT)	large	large	large	large	large	large	/	large	small	small	small	/	small		
Ventrolateral caudal fringe narrow or wide (VLF1)	wide	wide	wide	wide	wide	wide	/	wide	narrow	narrow	narrow	/	narrow		
Ventrolateral caudal fringe scales generally homogenous (VLF2)	no	no	no	no	no	no	/	no	no	no	no	/	no		
Tail cross-section (TLcross)	square	square	square	square	square	square	/	square	circular	circular	circular	/	circular		
Slightly enlarged medial subcaudals (SC1)	present	present	present	present	present	present	/	present	present	present	present	/	absent		
Single enlarged medial subcaudal (SC2)	absent	absent	absent	absent	absent	absent	/	absent	absent	absent	absent	/	absent		
Enlarged medial subcaudals intermittent, medially furrowed, posteriorly emarginate (SC3)	no	no	no	no	yes	no	/	no	no	no	no	/	no		
**Morphometric data**															
SVL	73.1	73.5	73.7	73.2	64.4	76.6	76.6	74.2	68.8	70.8	64.1	66.0	63.8		
AG	34.8	33.9	35.4	33.6	28.5	37.1	33.2	35.1	35.7	33.4	30.1	30.0	26.5		
HumL	8.4	7.2	9.0	9.0	7.2	8.0	8.1	8.6	9.7	9.3	8.0	9.6	9.5		
ForL	9.5	9.1	9.2	9.8	9.2	10.0	8.6	9.8	9.9	9.8	8.9	8.2	8.7		
FemL	12.8	11.6	12.3	12.5	10.9	13.7	10.8	12.5	12.0	12.6	11.5	11.7	9.8		
TibL	10.5	10.1	10.6	10.6	9.9	11.1	10.0	11.4	11.6	12.2	10.5	9.7	8.2		
HL	19.9	20.9	20.1	20.0	17.6	20.4	19.3	20.0	19.3	19.3	19.0	17.9	18.2		
HW	14.5	14.3	15.7	13.9	12.8	14.7	14.4	14.1	13.2	13.8	12.3	12.3	12.0		
HD	7.8	7.7	7.9	7.7	7.0	8.2	7.8	7.6	8.0	7.6	7.6	7.3	7.0		
ED	5.0	5.1	5.0	5.0	4.8	5.6	5.3	4.9	5.2	4.5	4.3	5.3	4.4		
EE	5.9	5.9	6.0	5.9	5.3	6.1	6.0	6.0	5.7	5.9	4.9	5.7	5.7		
ES	7.9	8.5	7.9	7.9	7.3	8.2	7.9	7.9	7.4	7.6	7.0	7.0	7.2		
EN	6.0	6.1	6.0	5.8	5.4	6.1	6.0	5.9	5.7	5.4	4.9	5.3	5.4		
IO	5.4	5.5	5.8	5.5	4.9	5.7	5.6	5.3	5.4	4.7	4.7	4.2	5.2		
EL	1.1	1.5	1.5	1.2	1.2	1.0	1.2	1.3	1.0	1.4	1.1	1.3	1.0		
IN	2.3	2.4	2.2	2.0	2.0	2.3	2.2	2.2	1.7	2.1	2.3	2.1	2.2		
**Species**	***C.elok***				***C.fluvicavus***							***C.interdigitalis***			
**Institutional catalog number**	**LSUHC 8238**	**LSUHC 12180**	**LSUHC 12181**	**ZMMU R-16144**	**ZMKU R 00959**	**ZMKU R 00958**	**ZMKU R 00960**	**ZMKU R 00961**	**ZMKU R 00962**	**ZMKU R 00963**	**ZMKU R 00964**	**THNHM 20226 paratype**	**THNHM 20228 paratype**		
**Sex**	♀	♂	♂	♀	♂	♂	♂	♀	♀	♀	♀	♀	♀		
**Meristic data**															
Supralabials (SL)	11	8	13	9	12R/12L	13R/12L	13R/12L	11R/12L	12R/12L	13R/12L	12R/11L	14	12		
Infralabials (IL)	11	8	11	9	10R/10L	10R/10L	9R/10L	10R/10L	10R/10L	10R/10L	10R/10L	9	8		
Paravertebral tubercles (PVT)	0	0	0	0	30	28	27	27	28	26	28	32	33		
Longitudinal rows of tubercles (LRT)	6	7	4	4	17	17	14	16	17	18	16	19	20		
Ventral scales (VS)	45	45	47	36	34	37	33	30	36	37	39	42	40		
Ventral scales along middle of the body (VSM)	190	225	234	192	155	154	155	172	164	175	170	187	170		
Expanded subdigital lamellae on 4^th^ toe (TL4E)	10	9	9	9	9R/9L	10R/10L	9R/9L	9R/9L	10R/11L	9R/10L	9R/9L	12	10		
Unmodified subdigital lamellae on 4^th^ toe (TL4U)	11	10	11	9	11R/11L	12R/11L	10R/10L	12R/12L	11R/11L	10R/10L	12R/13L	14	13		
Total subdigital lamellae 4^th^ toe (TL4T)	21	19	20	18	20R/20L	22R/21L	19R/19L	21R/21L	21R/22L	19R/20L	22R/22L	26	23		
Expanded subdigital lamellae on 4^th^ finger (FL4E)	8	9	9	9	8R/8L	8R/8L	8R/8L	8R/8L	7R/7L	8R/9L	7R/7L	9	8		
Unmodified subdigital lamellae on 4^th^ finger (FL4U)	12	13	9	8	10R/10L	10R/10L	10R/9L	11R/11L	10R/10L	9R/9L	10R/10L	12	11		
Total subdigital lamellae 4^th^ finger (FL4T)	20	22	18	17	18R/18L	18R/18L	18R/17L	19R/19L	17R/17L	17R/18L	17R/17L	21	21		
Enlarged femoral scales (R/L)	0	0	0	0	5R/6L	4R/5L	5R/6L	6R/6L	5R/6L	5R/6L	6R/6L	11R/8L	10R/9L		
Total enlarged femoral scales (FS)	0	0	0	0	11	9	11	12	11	11	12	14	19		
Total femoral pores in males (FP)	/	0	0	/	11	8	10	/	/	/	/	/	/		
Enlarged precloacal scales (PCS)	8	8	8	7	15	14	14	15	14	15	15	14	15		
Precloacal pores in males (PP)	/	8	8	/	15	14	14	/	/	/	/	/	/		
Postcloacal tubercles (PCT)	3	2	3	3	3R/2L	3R/2L	3R/3L	1R/1L	3R/2L	3R/3L	2R/2L	3	2		
Body bands (BB)	5	5	3	3	3	3	3	3	3	3	3	5	5		
**Categorical data**															
Small tubercles on flank (FKT)	absent	absent	absent	absent	present	present	present	present	present	present	present	present	present		
Dorsolateral caudal tubercles (DCT)	large	large	large	large	small	small	small	small	small	small	small	small	/		
Ventrolateral caudal fringe narrow or wide (VLF1)	wide	wide	wide	wide	narrow	narrow	narrow	narrow	narrow	narrow	narrow	narrow	/		
Ventrolateral caudal fringe scales generally homogenous (VLF2)	no	no	no	no	no	no	no	no	no	no	no	yes	yes		
Tail cross-section (TLcross)	square	square	square	square	circular	circular	circular	circular	circular	circular	circular	circular	/		
Slightly enlarged medial subcaudals (SC1)	absent	absent	absent	absent	present	present	present	present	present	present	present	absent	/		
**Species**	***C.elok***				***C.fluvicavus***							***C.interdigitalis***			
**Institutional catalog number**	**LSUHC 8238**	**LSUHC 12180**	**LSUHC 12181**	**ZMMU R-16144**	**ZMKU R 00959**	**ZMKU R 00958**	**ZMKU R 00960**	**ZMKU R 00961**	**ZMKU R 00962**	**ZMKU R 00963**	**ZMKU R 00964**	**THNHM 20226 paratype**	**THNHM 20228 paratype**		
**Sex**	♀	♂	♂	♀	♂	♂	♂	♀	♀	♀	♀	♀	♀		
Single enlarged medial subcaudal (SC2)	absent	absent	absent	absent	absent	absent	absent	absent	absent	absent	absent	absent	/		
Enlarged medial subcaudals intermittent, medially furrowed, posteriorly emarginate (SC3)	no	no	no	no	no	no	no	no	no	no	no	yes	/		
**Morphometric data**															
SVL	80.2	78.2	84.8	78.6	72.5	72.0	69.6	68.4	76.8	65.7	78.2	81.2	74.8		
AG	39.7	37.8	41.5	36.2	33.4	33.6	32.0	30.4	35.6	30.6	38.1	34.5	33.7		
HumL	10.2	9.1	10.1	1.7	9.1	8.8	9.0	8.0	10.0	7.5	10.1	9.8	10.2		
ForL	11.5	11.7	11.8	10.2	10.5	10.3	10.5	10.1	11.1	8.8	10.8	10.6	10.5		
FemL	12.9	14.2	14.6	13.1	13.1	12.5	12.5	13.5	14.1	11.5	13.9	14.7	13.2		
TibL	13.5	14.0	13.8	12.3	11.3	10.6	10.2	9.9	11.2	9.4	12.3	13.1	11.9		
HL	21.8	21.6	21.9	21.7	20.1	20.5	19.7	20.1	21.2	18.6	21.3	20.8	19.9		
HW	15.6	16.1	15.9	15.1	14.0	13.4	12.9	13.0	14.9	13.0	15.4	14.0	13.4		
HD	9.6	9.8	10.4	9.8	8.5	8.1	8.3	7.9	8.1	7.8	8.3	3.4	8.6		
ED	4.8	5.0	5.7	5.0	5.0	5.0	4.9	4.7	5.1	4.5	5.3	5.3	5.5		
EE	6.4	7.1	7.0	6.8	6.5	5.9	5.7	5.8	6.1	5.4	6.5	5.8	6.2		
ES	8.6	8.7	9.5	8.6	8.5	8.3	8.2	8.1	9.2	7.3	9.3	8.3	7.8		
EN	6.0	6.2	6.5	6.2	6.5	6.2	5.9	6.1	6.6	5.6	6.5	6.0	5.5		
IO	5.7	5.4	5.4	3.9	5.5	5.4	5.3	5.1	5.6	5.0	5.6	4.8	4.7		
EL	1.9	1.4	1.5	1.4	1.4	1.5	1.7	1.4	1.8	1.6	1.8	1.3	1.3		
IN	2.7	2.6	2.5	3.1	2.3	2.4	2.5	2.3	2.3	2.3	2.6	2.1	2.2		
**Species**	***C.kochangensis***	**C.cf.kochangensis**	***C.ngati***				***C.ngati*3**			***C.ngati*4**	**C.cf.ngati1**	**C.cf.ngati2**		***C.rivularis***	
**Institutional catalog number**	**ZMKU R 00945**	**THNHM 01667**	**HNUE-R00111**	**IEBR 4829**	**VNUF R.2020.12**	**HNUE-R00112**	**FMNH 255454**	**FMNH 270493**	**FMNH 270492**	**FMNH 265806**	**NCSM 79472**	**ZMMU R-14917**	**NCSM 80100**	**ZMKU R 00947**	**ZMKU R 00946**
**Sex**	♀	♂	♂	♀	♀	♀	♀	♂	♂	♂	♀	♀	♀	♀	♀
**Meristic data**															
Supralabials (SL)	12R/13L	12	10	10	10	10	13	13	13	10	14	9	12	13R/12L	13R/12L
Infralabials (IL)	9R/9L	10	9	9	9	9	10	9	11	8	11	10	12	11R/10L	10R/9L
Paravertebral tubercles (PVT)	34	29	39	40	38	40	28	27	26	27	28	32	29	34	33
Longitudinal rows of tubercles (LRT)	14	19	18	18	17	22	19	18	17	19	18	24	19	20	18
Ventral scales (VS)	35	34	38	36	35	32	37	36	36	33	33	36	35	34	37
Ventral scales along middle of the body (VSM)	172	159	168	164	178	158	159	166	156	158	164	166	165	160	166
**Species**	***C.kochangensis***	**C.cf.kochangensis**	***C.ngati***				***C.ngati*3**			***C.ngati*4**	**C.cf.ngati1**	**C.cf.ngati2**		***C.rivularis***	
**Institutional catalog number**	**ZMKU R 00945**	**THNHM 01667**	**HNUE-R00111**	**IEBR 4829**	**VNUF R.2020.12**	**HNUE-R00112**	**FMNH 255454**	**FMNH 270493**	**FMNH 270492**	**FMNH 265806**	**NCSM 79472**	**ZMMU R-14917**	**NCSM 80100**	**ZMKU R 00947**	**ZMKU R 00946**
**Sex**	♀	♂	♂	♀	♀	♀	♀	♂	♂	♂	♀	♀	♀	♀	♀
Expanded subdigital lamellae on 4^th^ toe (TL4E)	9R/8L	8	8	10	9	9	10	10	8	10	9	8	10	9R/9L	9R/9L
Unmodified subdigital lamellae on 4^th^ toe (TL4U)	12R/11L	13	11	10	11	10	11	11	11	11	12	10	10	13R/13L	12R/13L
Total subdigital lamellae 4^th^ toe (TL4T)	21R/19L	21	13	16	17	16	21	21	19	21	21	18	20	22R/22L	21R/22L
Expanded subdigital lamellae on 4^th^ finger (FL4E)	8R/8L	8	6	6	7	6	8	8	8	8	9	7	9	8R/8L	8R/8L
Unmodified subdigital lamellae on 4^th^ finger (FL4U)	10R/10L	12	9	9	9	9	10	10	10	10	8	9	10	11R/10L	12R/12L
Total subdigital lamellae 4^th^ finger (FL4T)	18R/18L	20	15	15	18	15	18	18	18	18	17	16	19	19R/18L	20R/20L
Enlarged femoral scales (R/L)	6R/6L	7R/7L	10R/10L	9R/8L	10R/9L	8R/9L	9R/7L	8R/9L	9R/9L	8R/8L	9R/8L	7R/8L	7R/8L	8R/8L	6R/8L
Total enlarged femoral scales (FS)	12	14	20	17	19	17	16	17	18	16	17	15	15	16	14
Total femoral pores in males (FP)	/	14	14	/	/	/	/	14	15	13	/	/	/	/	/
Enlarged precloacal scales (PCS)	12	16	13	13	13	13	15	13	13	13	12	13	13	15	15
Precloacal pores in males (PP)	/	16	/	/	/	/	13	13	13	13	/	/	/	/	/
Postcloacal tubercles (PCT)	1R/1L	3	3	2	1	2	0	0	0	0	2	3	4	2R/2L	3R/3L
Body bands (BB)	5	5	6	6	6	6	3	4	3	3	3	3	3	3	4
**Categorical data**															
Small tubercles on flank (FKT)	present	present	present	present	present	present	present	present	present	present	present	present	present	present	present
Dorsolateral caudal tubercles (DCT)	large	large	small	small	small	small	small	small	small	small	small	small	small	large	large
Ventrolateral caudal fringe narrow or wide (VLF1)	wide	wide	narrow	narrow	narrow	narrow	narrow	narrow	narrow	narrow	narrow	narrow	narrow	wide	wide
Ventrolateral caudal fringe scales generally homogenous (VLF2)	no	no	no	no	no	no	yes	yes	yes	yes	yes	yes	yes	yes	yes
Tail cross-section (TLcross)	square	/	circular	circular	circular	circular	circular	circular	circular	circular	circular	circular	circular	square	square
Slightly enlarged medial subcaudals (SC1)	present	present	present	present	present	present	/	present	present	present	present	present	present	absent	absent
Single enlarged medial subcaudal (SC2)	absent	absent	absent	absent	absent	absent	/	absent	absent	absent	absent	absent	absent	present	present
Enlarged medial subcaudals intermittent, medially furrowed, posteriorly emarginate (SC3)	no	no	no	no	no	no	/	no	no	no	no	no	no	no	no
**Morphometric data**															
SVL	60.1	70.2	66.5	68.1	69.3	46.6	83.6	70.2	74.1	73.8	78.0	87.1	77.7	73.9	68.1
**Species**	***C.kochangensis***	**C.cf.kochangensis**	***C.ngati***				***C.ngati*3**			***C.ngati*4**	**C.cf.ngati1**	**C.cf.ngati2**		***C.rivularis***	
**Institutional catalog number**	**ZMKU R 00945**	**THNHM 01667**	**HNUE-R00111**	**IEBR 4829**	**VNUF R.2020.12**	**HNUE-R00112**	**FMNH 255454**	**FMNH 270493**	**FMNH 270492**	**FMNH 265806**	**NCSM 79472**	**ZMMU R-14917**	**NCSM 80100**	**ZMKU R 00947**	**ZMKU R 00946**
**Sex**	♀	♂	♂	♀	♀	♀	♀	♂	♂	♂	♀	♀	♀	♀	♀
AG	29.0	31.5	28.8	29.8	30.2	19.7	41.3	35.4	37.0	31.3	38.2	41.9	36.8	34.8	33.2
HumL	6.5	10.2	7.9	8.1	8.5	5.6	8.6	8.7	8.6	6.9	8.7	11.5	9.2	8.1	7.6
ForL	7.6	8.6	9.2	10.0	10.1	6.5	10.2	9.3	10.4	10.0	10.3	10.4	10.7	9.7	9.1
FemL	10.4	12.1	11.5	11.5	11.5	7.6	13.7	12.7	13.0	13.1	13.1	15.2	14.2	11.4	10.4
TibL	8.4	11.8	10.8	11.1	11.8	7.8	12.5	11.8	11.2	11.1	12.8	12.6	12.7	11.2	10.3
HL	17.3	18.3	20.1	20.4	20.7	16.1	21.7	20.6	20.3	20.7	21.2	22.1	21.4	20.3	19.3
HW	11.6	12.1	12.6	12.0	11.8	8.8	13.8	12.5	13.0	12.3	12.7	14.8	13.5	14.9	13.7
HD	6.5	7.8	7.4	7.2	6.6	5.1	9.2	8.4	9.1	7.6	8.3	8.7	9.2	8.2	8.2
ED	4.2	5.2	3.8	4.1	3.4	2.6	4.9	4.9	4.9	4.8	6.5	4.6	6.0	5.8	5.6
EE	5.0	4.9	5.8	5.5	5.9	4.4	6.9	6.1	6.2	5.7	5.3	6.5	6.2	6.5	6.2
ES	6.9	7.5	7.5	7.6	6.9	5.0	9.0	8.3	8.3	8.2	8.7	8.8	8.4	8.3	7.9
EN	5.2	5.5	6.7	6.3	6.2	4.5	6.5	6.2	6.1	6.2	6.2	6.6	6.0	6.1	5.8
IO	4.2	4.0	5.6	5.4	5.6	4.2	6.6	5.6	5.4	5.1	4.9	3.5	5.7	5.8	5.5
EL	1.0	1.3	0.8	0.8	0.7	0.3	1.3	1.1	1.2	1.0	1.5	1.2	0.9	1.1	1.1
IN	1.9	2.2	2.8	2.6	2.6	2.0	2.8	2.5	2.5	2.3	2.7	2.2	2.5	2.3	2.0
**Species**	***C.rukhadeva***			**C.cf.rukhadeva**							***C.* sp. 11**	***C.* sp. 13**	***C.* sp. 13**	***C.uthaiensis***	
**Institutional catalog number**	**ZMMU R-16851**	**ZMMU R-16852**	**ZMKU R 00948**	**THNHM 24622**	**THNHM 24838**	**THNHM 03251**	**THNHM 03252**	**THNHM 03253**	**THNHM 03254**	**THNHM 01807**	**ZMMU R-16492**	**THNHM 00104**	**THNHM 27821**	**ZMKU R 00949**	
**Sex**	♂	♀	♀	♂	♀	♂	♂	♀	♂	♂	♂	♀	♀	♂	
**Meristic data**															
Supralabials (SL)	11	9	14	11	13	13	11	12	13	12	11	12	15	13R/15L	
Infralabials (IL)	10	11	9	10	10	10	10	10	11	10	9	10	11	10R/11L	
Paravertebral tubercles (PVT)	27	30	30	26	28	27	27	30	30	26	30	33	29	33	
Longitudinal rows of tubercles (LRT)	19	20	19	18	19	18	18	19	19	19	18	18	20	17	
Ventral scales (VS)	34	43	38	38	36	37	37	39	34	35	34	37	36	36	
Ventral scales along middle of the body (VSM)	154	152	165	162	158	157	159	168	160	161	160	159	165	159	
Expanded subdigital lamellae on 4^th^ toe (TL4E)	9	9	9	8	9	9	10	9	10	10	9	9	7	8R/(broken)L	
Unmodified subdigital lamellae on 4^th^ toe (TL4U)	11	11	12	11	13	12	12	15	13	13	10	12	12	12R/(broken)L	
Total subdigital lamellae 4^th^ toe (TL4T)	20	18	21	19	22	21	22	14	23	23	19	21	19	20	
Expanded subdigital lamellae on 4^th^ finger (FL4E)	9	8	8	7	8	8	8	8	8	8	10	8	8	7R/7L	
**Species**	***C.rukhadeva***			**C.cf.rukhadeva**							***C.* sp. 11**	***C.* sp. 13**	***C.* sp. 13**	***C.uthaiensis***	
**Institutional catalog number**	**ZMMU R-16851**	**ZMMU R-16852**	**ZMKU R 00948**	**THNHM 24622**	**THNHM 24838**	**THNHM 03251**	**THNHM 03252**	**THNHM 03253**	**THNHM 03254**	**THNHM 01807**	**ZMMU R-16492**	**THNHM 00104**	**THNHM 27821**	**ZMKU R 00949**	
**Sex**	♂	♀	♀	♂	♀	♂	♂	♀	♂	♂	♂	♀	♀	♂	
Unmodified subdigital lamellae on 4^th^ finger (FL4U)	10	9	11	10	11	10	10	12	12	12	9	11	10	11R/11L	
Total subdigital lamellae 4^th^ finger (FL4T)	19	17	19	17	17	18	18	20	20	20	19	19	18	18R/18L	
Enlarged femoral scales (R/L)	9R/8L	8R/8L	9R/8L	9R/L	9R/9L	9R/7L	7R/7L	6R/7L	5R/8L	7R/7L	9R/8L	9R/9L	7R/10L	8R/8L	
Total enlarged femoral scales (FS)	17	16	17	18	18	16	14	13	13	14	17	18	17	16	
Total femoral pores in males (FP)	17	/	/	14	/	12	13	/	11	13	17	/	/	12	
Enlarged precloacal scales (PCS)	17	13	15	15	15	14	13	15	15	14	13	14	16	14	
Precloacal pores in males (PP)	17	/	/	15	/	14	13	/	15	14	13	/	/	14	
Postcloacal tubercles (PCT)	3	2	2R/3L	3	2	3	2	2	3	2	3	3	3	3R/3L	
Body bands (BB)	3	3	3	3	3	4	4	/	/	5	3	3	/	6	
**Categorical data**															
Small tubercles on flank (FKT)	present	present	present	present	present	present	present	present	present	present	present	present	present	present	
Dorsolateral caudal tubercles (DCT)	small	small	small	small	small	small	small	small	small	/	large	small	small	large	
Ventrolateral caudal fringe narrow or wide (VLF1)	narrow	narrow	narrow	narrow	narrow	narrow	narrow	narrow	narrow	/	wide	narrow	narrow	wide	
Ventrolateral caudal fringe scales generally homogenous (VLF2)	yes	yes	yes	yes	yes	yes	yes	yes	yes	/	yes	yes	yes	no	
Tail cross-section (TLcross)	square	square	square	square	square	square	square	square	square	/	square	circular	circular	circular	
Slightly enlarged medial subcaudals (SC1)	absent	absent	absent	absent	absent	absent	absent	absent	absent	/	present	present	present	present	
Single enlarged medial subcaudal (SC2)	present	present	present	present	present	present	present	present	present	/	absent	absent	absent	absent	
Enlarged medial subcaudals intermittent, medially furrowed, posteriorly emarginate (SC3)	no	no	no	no	no	no	no	no	no	no	no	no	no	yes	
**Morphometric data**															
SVL	74.9	71.7	71.6	68.3	71.8	73.6	75.3	74.7	73.2	61.5	68.1	63.7	72.9	58.1	
AG	34.6	32.6	33.9	27.3	29.9	30.9	31.3	32.2	30.3	26.2	34.6	25.8	30.6	26.6	
HumL	10.7	10.4	7.9	9.8	8.3	12.2	11.3	11.8	11.0	10.1	10.3	7.6	10.1	7.0	
ForL	8.6	7.9	9.6	8.7	8.5	9.0	10.6	9.6	9.2	7.9	8.5	8.1	9.6	8.3	
FemL	12.6	11.8	10.5	10.8	10.9	11.5	10.2	11.9	12.1	9.5	12.6	10.7	12.8	10.0	
TibL	10.1	9.3	11.2	9.7	10.7	10.9	11.7	11.3	11.1	9.1	11.4	10.1	10.2	8.4	
HL	20.2	19.2	19.7	19.7	19.9	20.8	21.3	20.8	21.5	17.9	18.4	17.6	19.9	16.1	
HW	14.6	13.4	14.0	13.1	13.9	14.9	15.0	13.1	14.1	11.8	13.1	11.9	13.8	10.9	
**Species**	***C.rukhadeva***			**C.cf.rukhadeva**							***C.* sp. 11**	***C.* sp. 13**	***C.* sp. 13**	***C.uthaiensis***	
**Institutional catalog number**	**ZMMU R-16851**	**ZMMU R-16852**	**ZMKU R 00948**	**THNHM 24622**	**THNHM 24838**	**THNHM 03251**	**THNHM 03252**	**THNHM 03253**	**THNHM 03254**	**THNHM 01807**	**ZMMU R-16492**	**THNHM 00104**	**THNHM 27821**	**ZMKU R 00949**	
**Sex**	♂	♀	♀	♂	♀	♂	♂	♀	♂	♂	♂	♀	♀	♂	
HD	9.2	8.5	8.3	7.3	8.9	8.2	8.2	8.1	8.9	7.5	8.3	7.7	8.4	6.3	
ED	4.6	4.3	5.5	4.9	5.1	5.8	5.4	5.0	5.5	4.7	4.4	4.1	5.3	4.6	
EE	6.2	6.2	5.8	5.1	6.2	5.6	5.7	5.4	6.2	4.3	6.2	4.9	6.3	4.7	
ES	8.3	7.7	7.9	7.4	8.1	8.4	8.8	8.1	8.6	7.3	7.7	7.2	8.0	6.4	
EN	6.3	5.7	5.8	5.4	6.0	6.2	6.4	5.8	6.2	5.3	5.5	5.6	5.9	4.9	
IO	3.3	3.1	5.6	4.5	4.7	5.6	5.7	5.7	5.6	4.2	2.9	4.8	6.1	4.3	
EL	1.2	1.0	1.4	1.6	1.5	1.2	1.3	1.2	1.2	0.9	0.9	1.4	1.4	1.5	
IN	2.2	2.1	2.1	2.0	2.2	2.4	2.5	2.4	2.3	2.0	2.3	2.1	2.3	1.8	

#### Description of holotype

**(Fig. 4).** Adult male SVL 73.2 mm; head moderate in length (HL/SVL 0.27), width (HW/HL 0.70), depth (HD/HL 0.39), distinct from neck, triangular in dorsal profile; lores concave slightly anteriorly, weakly inflated posteriorly; prefrontal region concave; canthus rostralis rounded; snout elongate (ES/HL 0.40), rounded in dorsal profile; eye large (ED/HL 0.25); ear opening horizontally elliptical, small; eye to ear distance greater than diameter of eye; rostral rectangular, divided by a dorsal furrow, bordered posteriorly by large left and right supranasals and one small azygous internasal, bordered laterally by first supralabials; external nares bordered anteriorly by rostral, dorsally by large supranasal, posteriorly by two unequally sized smaller postnasals, bordered ventrally by first supralabial; 14R/14L rectangular supralabials, second through eighth supralabials nearly same size as first, then tapering below eye; 10R/10L infralabials tapering smoothly to just below and slightly past posterior margin of eye; scales of rostrum and lores flat to slightly domed, larger than granular scales on top of head and occiput; scales of occiput intermixed with distinct, small tubercles; superciliaries subrectangular, largest anterodorsally; mental triangular, bordered laterally by first infralabials and posteriorly by large left and right trapezoidal postmentals contacting medially for 45% of their length posterior to mental; one row of enlarged, square to rectangular sublabials extending posteriorly to sixth(L) and fifth(R) infralabial; gular and throat scales small, granular, grading posteriorly into slightly larger, flatter, smooth, imbricate, pectoral and ventral scales.

Body relatively short (AG/SVL 0.46) with well-defined ventrolateral folds; dorsal scales small, granular interspersed with larger, conical, semi-regularly arranged, weakly keeled tubercles; tubercles extend from occipital region onto base of tail and slightly beyond as paravertebral rows; smaller tubercles extend anteriorly onto nape and occiput, diminishing in size anteriorly; approximately 20 longitudinal rows of tubercles at midbody; approximately 34 paravertebral tubercles; tubercles on flanks; 34 longitudinal rows of flat, imbricate, ventral scales much larger than dorsal scales; 166 transverse rows of ventral scales; 15 large, pore-bearing, precloacal scales; no deep precloacal groove or depression; and two rows of enlarged post-precloacal scales on midline.

Forelimbs moderate in stature, relatively short (ForL/SVL 0.13); granular scales of forearm larger than those on body, interspersed with large flat tubercles; palmar scales rounded, slightly raised; digits well-developed, relatively short, inflected at basal interphalangeal joints; digits narrower distal to inflections; subdigital lamellae wide, transversely expanded proximal to joint inflections, narrower transverse lamellae distal to joint inflections; claws well-developed, claw base sheathed by a dorsal and ventral scale; 8R/8L expanded and 11R/11L unexpanded lamellae beneath the fourth finger; hind limbs larger and thicker than forelimbs, moderate in length (TibL/SVL 0.14), covered dorsally by granular scales interspersed with moderately sized, conical tubercles dorsally and posteriorly and anteriorly by flat, slightly larger, subimbricate scales; ventral scales of thigh flat, imbricate, larger than dorsals; subtibial scales flat, imbricate; one row of 6R/8L enlarged pore-bearing femoral scales not continuous with enlarged pore-bearing precloacal scales, terminating distally at knee; 7R/8L enlarged femoral scales; proximal femoral scales smaller than distal femorals, the former forming an abrupt union with much smaller, rounded, ventral scales of posteroventral margin of thigh; plantar scales flat, subimbricate; digits relatively long, well-developed, inflected at basal interphalangeal joints; 8R/8L wide, transversely expanded subdigital lamellae on fourth toe proximal to joint inflection extending onto sole, and 12R/12L unexpanded lamellae beneath the fourth toe distal to joint inflection; and claws well-developed, claw base sheathed by a dorsal and ventral scale.

Tail original, 94.6 mm long (TL/SVL 1.29), 5.0 mm in width at base, tapering to a point; nearly square in cross-section; dorsal scales flat, intermixed with tubercles forming paravertebral rows anteriorly and larger tubercles forming dorsolateral longitudinal rows; large, posteriorly directed, semi-spinose tubercles forming wide ventrolateral caudal fringe; larger scales of ventrolateral fringe occur at regular intervals; medial subcaudals enlarged but not paired, an enlarged single medial subcaudal longitudinal row absent; subcaudals, larger than dorsal caudals; base of tail bearing hemipenal swellings; 3R/3L conical postcloacal tubercles at base of hemipenal swellings; and postcloacal scales flat, imbricate.

#### Coloration in life

**(Fig. 4).** Ground color of the head body, limbs, and tail dull yellow; diffuse darker mottling on the top of the head; wider, pale-brown pre- and postorbital stripe extends from external nares to angle of jaw; whitish canthal and postorbital stripe dorsal to pale-brown pre- and postorbital stripe; faint, pale brown, nuchal band bearing two posteriorly directed projections; paired dark-brown paravertebral blotches on nape; four wide, irregularly shaped and broken transverse body bands edged in slightly pale brown between limb insertions; band interspaces bearing irregularly shaped scattered pale-brown markings; very faint pale-brown speckling on limbs and digits; seven wide pale-brown caudal bands separated by seven paler colored bands; posterior five pale-brown caudal bands encircle tail; ventral surfaces of body and limbs beige, generally immaculate, subcaudal region generally darker; iris orange-gold in color bearing black vermiculations.

#### Variation

**(Fig. 5, Table 5).** Individuals of the type series are very similar in overall coloration and pattern. TL and TW of complete original tails (ZMKU R 00951–00952, ZMKU R 00954, ZMKU R 00957) are 80.1–94.7 mm (mean 89.1 ± 6.5 mm; *N* = 4) and 4.2–4.9 mm (mean 4.7 ± 0.3; *N* = 4), respectively. ZMKU R 00956 has a short, partially regenerated tail which lacks banding (TL 27.7 mm, TW 5.1 mm). Similarly, the posterior sections of the tails in ZMKU R 00950 (TL 75.5 mm, TW 5.0 mm) and ZMKU R 00955 (TL 73.3 mm, TW 4.7 mm) are regenerated. Specimens ZMKU R 00950, ZMKU R 00952, and ZMKU R 00954 have three as opposed to four body bands in the holotype and ZMKU R 00955 has five body bands. Raw morphometric and meristic differences within and among all species of the *brevipalmatus* group are listed in Table 5.

#### Distribution.

*Cyrtodactylusthongphaphumensis* sp. nov. is currently known only from the type locality at Thong Pha Phum National Park, Pilok Subdistrict, Thong Pha Phum District, Kanchanaburi Province, Thailand (Fig. 1).

#### Etymology.

The specific epithet *thongphaphumensis* is in reference to the type locality of Thong Pha Phum National Park.

#### Comparisons.

*Cyrtodactylusthongphaphumensis* sp. nov. is the sister species to a clade composed of eight lineages in the phylogenetic sequence of *C.uthaiensis*, sp. 11, *C.interdigitalis*, C.cf.ngati1, C.cf.ngati2, *C.ngati*3, and the sister lineages *C.ngati*4 and *C.ngati* (Fig. 2). *Cyrtodactylusthongphaphumensis* sp. nov. differs from those lineages by an uncorrected pairwise sequence divergence of 7.6–9.7% and from all members of the *brevipalmatus* group by 7.6–22.3% (Table 2). It differs discretely from *C.elok* by having as opposed to lacking paravertebral tubercles, femoral and precloacal pores, and by having 19–21 as opposed to 4–7 longitudinal rows of tubercles. It differs from *C.brevipalmatus*, *C.fluvicavus*, *C.interdigitalis*, *C.ngati*, *C.ngati*3, and *C.rukhadeva* in having statistically significant different mean values of combinations of the morphometric characters of AG, HumL, ForL, TibL, HL, HW, HD, EE, ES, EN, EL, and IN (Table 3). It differs further from those same species in having statistically significant different mean values of combinations of the meristic characters SL, PVT, LRT, VS, VSM, TL4T, FL4E, FL4U, FL4T, FS, PCS, and BB (Table 3). Discrete differences between *Cyrtodactylusthongphaphumensis* sp. nov. and other putative species and populations are presented in Table 5.

#### Natural history.

**Figure 6.**
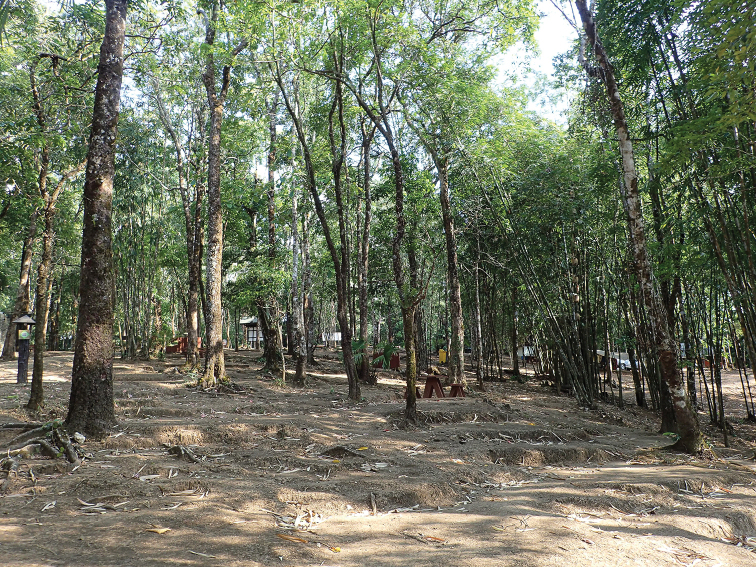
Habitat of the type locality at Thong Pha Phum National Park, Pilok Subdistrict, Thong Pha Phum District, Kanchanaburi Province, Thailand.

All individuals were found in hill evergreen forest at 914 m elevation (Fig. 6). Specimens (*N* = 8) were collected at night (1900–2100 h) during the dry season (April) on tree trunks (62.5%; *N* = 5), on a building (12.5%; *N* = 1), and the ground (25.0%; *N* = 2) with a temperature of 27.0 °C and relative humidity of 71.1%. The holotype (ZMKU R 00953) and four paratypes (ZMKU R 00950, ZMKU R 00954, ZMKU R 00956–00957) were found on tree trunks ≤ 160 cm above ground level. One specimen (ZMKU R 00951) was found on a building. Two specimens (ZMKU R 00952, ZMKU R 00955) were found on ground. At night, the new species was found to co-occur with other gekkonid lizards, *Cyrtodactylusoldhami* (Theobald, 1876), *Gekkokaengkrachanense* (Sumontha, Pauwels, Kunya, Limlikhitaksorn, Ruksue, Taokratok, Ansermet & Chanhome, 2012), and *Hemidactylusgarnotii* Duméril & Bibron, 1836.

## ﻿Discussion

The discovery of new populations of the *Cyrtodactylusbrevipalmatus* group across the archipelago of the upland sky-island habitats in Thailand will likely be commonplace with increased field work. Many such undescribed populations have already been reported and photographed on social networking platforms and these populations will be sampled and analyzed in order to ascertain their species status. Grismer et al. (2022c) pointed out that for several years many such populations went unanalyzed and were simply placed in the synonymy of either *C.brevipalmatus* or *C.interdigitalis*, only to be elevated later to species status following data-rich phylogenetic delimitation and morphological diagnostic analyses (Grismer et al. 2021c, 2022c). This current work not only contributes to an increased understanding of the unrealized diversity within the *brevipalmatus* group, but to a growing body of literature underscoring the high degree of herpetological diversity and endemism across a sky-island archipelago of upland montane tropical forests in Thailand (see Suwannapoom et al. 2022) which like many other upland tropical landscapes, are becoming some of the most imperiled ecosystems on the planet.

## Supplementary Material

XML Treatment for Cyrtodactylus thongphaphumensis

## Supplementary materials

Supplementary material 1 Data frame for the multiple factor analysis of the putative species of the *Cyrtodactylusbrevipalmatus* group Data type morphological data https://binary.pensoft.net/file/797566This dataset is made available under the Open Database License (http://opendatacommons.org/licenses/odbl/1.0/). The Open Database License (ODbL) is a license agreement intended to allow users to freely share, modify, and use this Dataset while maintaining this same freedom for others, provided that the original source and author(s) are credited.L. Lee Grismer, Attapol Rujirawan, Siriwadee Chomdej, Chatmongkon Suwannapoom, Siriporn Yodthong, Akrachai Aksornneam, Anchalee Aowphol
